# Sterol Composition of Clinically Relevant Mucorales and Changes Resulting from Posaconazole Treatment

**DOI:** 10.3390/molecules23051218

**Published:** 2018-05-19

**Authors:** Christoph Müller, Thomas Neugebauer, Patrizia Zill, Cornelia Lass-Flörl, Franz Bracher, Ulrike Binder

**Affiliations:** 1 Department of Pharmacy-Center for Drug Research, Ludwig-Maximilians University of Munich, Butenandtstr. 5-13, 81377 Munich, Germany; christoph.mueller@cup.uni-muenchen.de (C.M.); patrizia.zill@campus.lmu.de (P.Z.); franz.bracher@cup.uni-muenchen.de (F.B.); 2 Department of Hygiene, Microbiology and Public Health, Division of Hygiene and Medical Microbiology, Medical University Innsbruck, Schöpfstr. 41, 6020 Innsbruck, Austria; tneugebauer@gmx.at (T.N.); cornelia.lass-floerl@i-med.ac.at (C.L.-F.)

**Keywords:** Mucorales, *Rhizopus arrhizus*, sterol pattern, antifungal effectivity, gas chromatography-mass spectrometry (GC-MS), posaconazole

## Abstract

Mucorales are fungi with increasing importance in the clinics. Infections take a rapidly progressive course resulting in high mortality rates. The ergosterol biosynthesis pathway and sterol composition are of interest, since they are targeted by currently applied antifungal drugs. Nevertheless, Mucorales often exhibit resistance to these drugs, resulting in therapeutic failure. Here, sterol patterns of six clinically relevant Mucorales (*Lichtheimia corymbifera*, *Lichtheimia ramosa*, *Mucor circinelloides*, *Rhizomucor pusillus*, *Rhizopus arrhizus*, and *Rhizopus microsporus*) were analysed in a targeted metabolomics fashion after derivatization by gas chromatography-mass spectrometry. Additionally, the effect of posaconazole (POS) treatment on the sterol pattern of *R. arrhizus* was evaluated. Overall, fifteen different sterols were detected with species dependent variations in the total and relative sterol amount. Sterol analysis from *R. arrhizus* hyphae confronted with sublethal concentrations of posaconazole revealed the accumulation of 14-methylergosta-8,24-diene-3,6-diol, which is a toxic sterol that was previously only detected in yeasts. Sterol content and composition were further compared to the well-characterized pathogenic mold *Aspergillus fumigatus*. This work contributes to a better understanding of the ergosterol biosynthesis pathway of Mucorales, which is essential to improve antifungal efficacy, the identification of targets for novel drug design, and to investigate the combinatorial effects of drugs targeting this pathway.

## 1. Introduction

**Figure 1:**
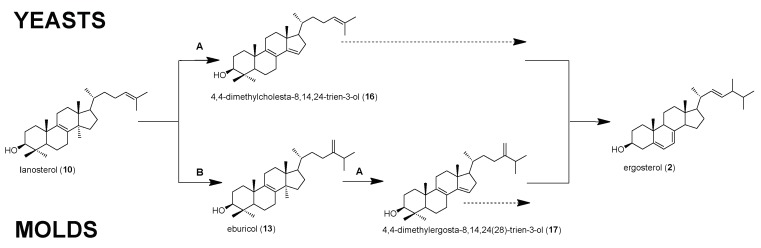
Preferred ergosterol biosynthesis pathways in yeasts (*S. cerevisiae*) and molds (*A. fumigatus*) starting from lanosterol. Enzymes: (**A**) sterol C14-demethylase, (**B**) sterol C24-methyltransferase.

The order Mucorales represent the most prominent order of zygospore-forming fungi, which was formerly placed in the phylum Zygomycota and was referred to as Zygomycetes. Over the years, this phylum has undergone constant taxonomic rearrangements. Now, the Mucorales, or mucormycetes, are placed in the phylum Glomeromycota and subphylum Mucormycotina [1,2,3]. Mucorales are mainly saprophytes commonly found in soil and decomposing material. Members of this group play various important roles for human life—e.g., *Mucor* spp., are of biotechnological importance due to their high growth rates and their great potential in the production of secondary metabolites [4]. Other Mucorales, such as *Rhizopus* spp., have been used for food fermentation in Asia for centuries [5]. Contrary to these positive effects of mucoralean fungi on human life, they also cause a wide range of diseases to plants, animals, and human beings. In the last ten years, the number of mucormycosis cases (also known as zygomycosis) [3,6] has increased significantly in the clinics. Mucorales that were isolated in high abundance from patient material belong to the following genera: *Rhizopus*, *Lichtheimia* (formerly *Absidia*), *Mucor*, *Rhizomucor,* and *Cunninghamella*. Worldwide, *Rhizopus arrhizus* is the most common species that was isolated from clinical specimen. The clinical presentation of mucormycosis varies, with mostly rhinocerebral, pulmonary and gastrointestinal manifestations. Primarily, immunocompromised patients are at risk to develop mucormycosis, but some forms, such as cutaneous mucormycosis, have also been seen in otherwise healthy persons [1,7]. The aggressive course of the disease, delayed diagnosis, and poor treatment options result in unacceptably high mortality rates (40–70%), even with antifungal therapy. Often, mucormycosis is seen as so called breakthrough infection when voriconazole is applied as prophylactic treatment regime [6]. Antifungal drugs currently licensed for treatment of mucormycosis are the polyene antifungal amphotericin B (AMB), preferably the liposomal formulation as first line treatment, and the triazole posaconazole as salvage treatment. In 2015, another azole, isavuconazole (ISA), was approved by the Food and Drug Administration (FDA) as a stand-alone treatment for mucormycosis [1,8,9,10,11,12,13]. AMB forms 1:1 adducts with fungal ergosterol and induces an accumulation of reactive oxygen species in the cytoplasm [14,15]. Azoles conduct their antifungal activity by inhibiting the enzyme sterol C14-demethylase (named ERG11 in yeasts, and CYP51 in *Aspergillus* spp., Figure 1, enzyme **A**) [16,17]. POS is also very active against *Aspergillus* isolates and used in the therapy of invasive aspergillosis. In contrast, another second-generation triazole, voriconazole (VRC), was shown to have no in vitro nor in vivo activity against Mucormycetes [13,15,18,19,20]. The limited arsenal of highly active anti-mucoralean drugs, plus the occurrence of AMB and azole resistant strains highlight the importance of illuminating the mode of action of antifungal drugs on, and resistance mechanisms in this group of fungi [13,15]. As the target of the currently used antifungal drugs against Mucorales is ergosterol or its biosynthesis, respectively, it is of great importance to understand the biosynthetic pathway and decipher the differences to other human pathogenic fungi.

For two major human pathogenic fungi, the yeast *Candida albicans*, and the mold *Aspergillus fumigatus*, differences in the ergosterol biosynthesis pathways have already been elucidated. In *Candida* spp., and this is similar to baker´s yeast *S. cerevisiae* (Figure 1, upper panel), lanosterol is the preferred substrate for the sterol C14-demethylase (Figure 1, enzyme **A**), which converts lanosterol into 4,4-dimethylcholesta-8,14,24-trien-3β-ol [21,22,23,24,25]. In molds, e.g., *Aspergillus fumigatus*, the preferred ergosterol biosynthesis route from lanosterol starts with a methylation at C-24 by sterol C24-methyltransferase (enzyme **B**) in order to give eburicol, and is then followed by a demethylation at C-14 by sterol C14-demethylase to give 4,4-dimethylergosta-8,14,24(28)-trien-3β-ol [24,25,26] (Figure 1, lower panel).

In the presence of azoles, the accumulation of physiological substrates for sterol C14-demethylase and a depletion of ergosterol are observed in both yeasts and molds [16,25,27,28,29,30]. The accumulation of C14-methylated sterols (e.g., lanosterol (**10**) and eburicol (**13**)) and lower levels of ergosterol as the predominant native sterol in fungi or changes in the relative sterol composition results in alterations of the plasma membrane that impact its function and the activity of membrane-bound enzymes [31,32,33].

So far, very little is known about the ergosterol biosynthesis pathway, the sterol content, and the composition of clinically relevant Mucorales, and most studies were carried out with one species or one strain only, which makes the comparison in-between the group of Mucorales difficult [26,34,35,36,37,38,39]. Therefore, a full understanding of the ergosterol biosynthesis pathway and the sterol pattern of this group of pathogenic fungi is essential to determine the efficacy of antifungal therapy, to identify targets for novel drug design, and to investigate the combinatorial effects of drugs targeting the ergosterol biosynthesis pathway. Here, the sterol pattern and sterol content of six clinically relevant Mucorales (*Rhizopus (R.) arrhizus*, *Rhizopus microsporus Lichtheimia (L.) corymbifera*, *Lichtheimia ramosa*, *Mucor (M.) circinelloides,* and *Rhizomucor (Rh.) pusillus*) were analysed in a targeted metabolomics fashion by gas chromatography-mass spectrometry (GC-MS). In addition, sterols in hyphae of *R. arrhizus* that was confronted with sub-lethal concentrations of posaconazole were analysed and compared to untreated hyphae. *R. arrhizus* was chosen for this test because it represents the most abundant Mucorales species from patient material worldwide. Furthermore, the obtained data were compared to the well-studied ergosterol biosynthetic pathway of *Aspergillus fumigatus* [16,24,25,27,30,40,41,42,43,44,45,46].

## 2. Results and Discussion

### 2.1. Relative and Absolute Amounts of Sterol Intermediates in Mucorales

**Figure 2:**
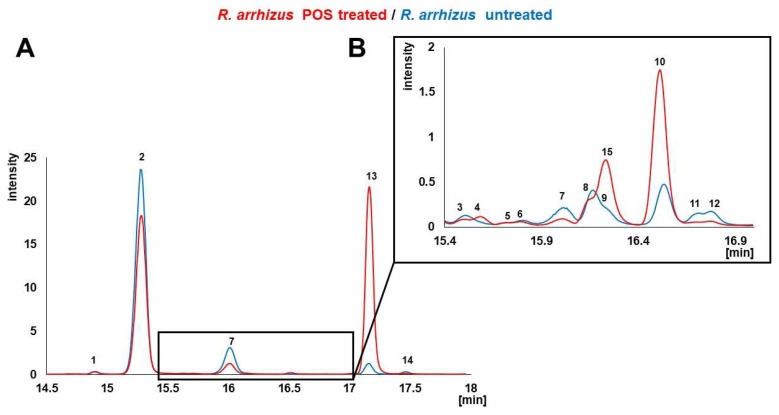
Representative selected ion chromatograms of untreated *R. arrhizus* samples (blue) and hyphae confronted with posaconazole (red). Hyphae were confronted with sublethal concentrations (0.5 µg/mL) for 4 h. Numbers in the diagram represent the sterol intermediates as given in Table 1. X-axis presents retention time. Selected ions for chromatogram (**A**) *m*/*z* 363 + 366 + 365 + 407 + 408 and selected ions for chromatogram (**B**) *m*/*z* 343 + 377 + 379 + 393 + 472 + 486.

In total, 14 different sterols were detected (Table 1) in the six investigated *Mucormycetes* (without azole treatment), which are discussed in the order of their retention time below (Figure 2, blue chromatogram).

Ergosta-5,8,22-trien-3β-ol (lichesterol, **1**) was detected in all of the six species tested. Among the Mucormycetes, highest relative amounts of this intermediate were detected in *Rhizopus* spp., although these amounts were still lower than in *A. fumigatus*, which was included for comparison.

As expected, ergosta-5,7,22-trien-3β-ol (ergosterol, **2**) was identified as the dominating sterol in all the species studied. This is in agreement with a study that was carried out with *R. arrhizus* [39], in which ergosterol was also identified as the sterol exhibiting highest abundance. This finding is supported by a study that was investigating the phylogenetic distribution of fungal sterols [39]. One contrary result was shown by Weete et al. [37], explaining that ergosta-7,22-dien-3β-ol (**3**) is the major sterol (56.0 to 59.9%) and ergosterol (**2**) is only the second most abundant sterol (21.1–28.4%) in *R. arrhizus*. Importantly, the relative content of ergosterol was highly variable between the species, ranging from 38.0% in *Rh. pusillus* to 76.3% in *R. arrhizus*. In *A. fumigatus,* the relative amount of ergosterol reached 95.0% of all the sterols in our experimental setup. This amount is slightly higher than what was shown by a comprehensive study of Alcazar-Fuoli et al. [24], where *A. fumigatus* isolates reached relative ergosterol amounts of 75.8 to 88.4%. Differences in experimental setup, like growth media and the age of the cultures used for sterol extraction, might explain these differences.

Only minor amounts of ergosta-7,22-dien-3β-ol (**3**) were detected in most species. Only *L. corymbifera* and *Rh. pusillus* showed little higher accumulation of **3**, representing 3.5% and 1.9%, respectively, whereas this intermediate was neither detected in *M. circinelloides* nor in *A. fumigatus*.

The relative amount of ergosta-5,7,22,24(28)-tetraen-3β-ol (**4**) and ergosta-7,22,24(28)-trien-3β-ol (**5**) was very low in all of the samples (0.2–1.2%). This is in agreement with the value of 1.2% of **4** reported for *M. rouxii* [36].

Sterol **6**, ergosta-5,7,24(28)-trien-3β-ol, was found in five out of the seven species tested (*A. fumigatus* included), it exhibited the third highest abundancy among all the sterols in *L. ramosa* (7.4%) and *M. circinelloides* (10.4%). Interestingly, sterol **6** was missing in *L. corymbifera* and *Rh. pusillus*.

In all Mucorales strains, significant amounts of ergosta-5,7-dien-3β-ol (**7**), the saturated side chain analogue of ergosterol (**2**) were found (10.6–37.2%), making it the second most abundant sterol after ergosterol in the Mucorales, whereas only a small amount (0.3%) of this sterol was detected in *A. fumigatus*. Interestingly, in *Rh. pusillus* the relative amount of ergosterol (**2**) and ergosta-5,7-dien-3β-ol (**7**) were nearly equal (38.0% and 37.2%), a characteristic that seems unique for this species. McCorkindale et al. [34] also detected larger amounts of **7** in Mucorales (mean 45%; n = 8) and also Weete et al. [39] identified **7** in *Rhizomucor pusillus* (formerly named *Mucor pusillus*). This indicates a putative role of ergosta-5,7-dien-3β-ol (**7**) as a marker sterol for the identification and classification of Mucorales species. This sterol was not found in studies evaluating sterols of *A. fumigatus* [24,40], while we detected minor amounts (0.3%) in our cultures. From previous studies, we learned that ergosta-5,7-dien-3β-ol (**7**) is only found in very little amounts in molds [25,41,42] and yeasts [25,29,43,44].

Episterol, ergosta-7,24(28)-dien-3β-ol (**8**), was evident in all of the samples, ranging from 1.4% (*Rh. pusillus*) to 2.4% in (*L. corymbifera* and *R. microsporus*). This sterol was also detected by Safe [36] in another mucoralean fungus, *M. rouxii*, in which the amount of free and bounded **8** was shown to be strongly dependent on the growth conditions (1.1–25.8%).

All of the Mucorales strains produced ergost-7-en-3β-ol (**9**) with maximum levels in *L. corymbifera* (3.7%) and *Rh. pusillus* (3.0%). In contrast to the results of Weete et al. [37] (mean 13.5%; n = 6), we detected clearly lower levels (0.3%) of **9** in *R. arrhizus*.

Low percentages (≤ 1.3%) of lanosterol (4,4,14-trimethylcholesta-8,24-dien-3β-ol, **10**), T-MAS (4,4-dimethylcholesta-8,24-dien-3β-ol, **11**), and 4-methylergost-8-en-3β-ol (**12**) were ubiquitously found in all of the samples.

Most interestingly, a high amount of eburicol (4,4,14-trimethylergosta-8,24(28)-dien-3β-ol, **13**) was detected to a degree that is usually only observed under azole treatment in fungi [25,29,41,43]. Levels reached up to 15.4% in *Rh. pusillus*, all other species also showed considerably high eburicol levels, ranging from 2.0% (*L. corymbifera*) to 7.2% (*M. circinelloides*). In our experimental setup, we did not detect eburicol in *A. fumigatus*, which is in contrast to the sterol profile of *A. fumigatus* in Alcazar-Fuoli et al. [24], where an eburicol content of approx. 1.9% was reported. Different growth conditions and different growth media might explain this discrepancy.

The last detected sterol, 4,4-dimethylergosta-8,24(28)-dien-3β-ol (**14**), was present in all of the samples, ranging from 0.3% (*R. arrhizus*) to 1.1% (*M. circinelloides*).

Sterol **1** and the sterols **4**, **6**, **10**–**14** were not explicitly mentioned in previous reports on the sterol composition of Mucormycetes [26,34,35,36,37,38,39], but the percentages that we obtained in our study were in the range of other fungi [22,24,40,41,42].

Additional to the relative amount of sterol intermediates, we also determined the actual amount of each sterol, which was expressed as µg/mg biomass, (dry weight, Table 1). The total sterol content was proportionately 1–2% of the total mycelial biomass, which is in agreement with what has been detected for other *Mucor* species before [36]. Lowest amount of total sterols was found in *R. arrhizus* (9.26 µg/mg) and highest in *Rh. pusillus* (21.39 µg/mg). The values of each intermediate determined correlate with the relative amounts that were previously described. In *A. fumigatus,* a total sterol content of 9.78 µg/mg was determined, of which 9.25 µg/mg was ergosterol, an amount that is comparable to previously reported data [24].

### 2.2. Sterol Composition and Sterol Content of Posaconazole Treated R. arrhizus

**Figure 3:**
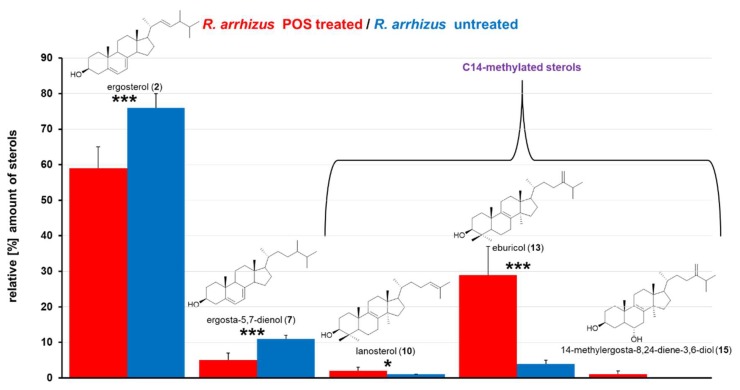
Relative amounts [%] of the most prominent sterols in posaconazole treated (red) and untreated (blue) *R. arrhizus* hyphae. Cultures, pre-grown for 16 h, were confronted with 0.5 µg/mL posaconazole for 4 h before sterol extraction. Sterol pattern was compared to untreated controls, which were incubated under identical conditions. Error bars represent standard deviation out of two independent experiments, comprising six technical replicates. (* *p* < 0.05; *** *p* < 0.001: student’s *t*-test). For detailed information on all sterols extracted, see Table 2.

To investigate the effect of the sterol C14-demethylase inhibitor POS on the sterol composition and content of Mucorales, *R. arrhizus* hyphae confronted with sublethal concentrations (0.5 µg/mL) of POS were compared to the sterol pattern of untreated hyphae Figure 2 and Figure 3, Table 2. For comparison and also to validate our results that were obtained with Mucorales, we included *A. fumigatus*, in which the effect of azole treatment on sterol pattern has been extensively studied [24,25,40,41,45].

**Figure 4:**
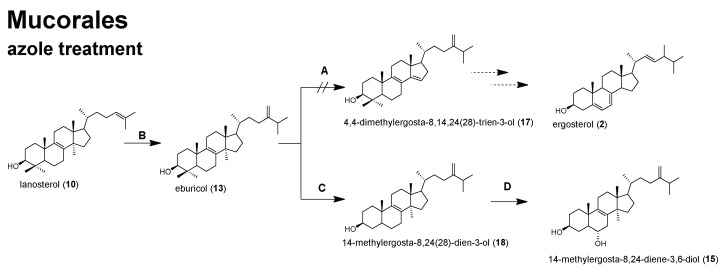
Putative alternative sterol biosynthesis pathway from lanosterol in Mucorales in the presence of posaconazole. Enzymes: (**A**) sterol C14-demethylase, (**B**) sterol C24-methyltransferase, (**C**) sterol C4-demethylase complex, (**D**) sterol C5-desturase.

As expected, the relative amount of ergosterol (**2**) was significantly reduced by 17.8% in *R. arrhizus* due to POS treatment. On the other hand, eburicol (**13**), which is one of the substrates of the azoles’ target enzyme C14-demethylase, increased by 25.4% (Figure 3), which underlines the enzyme inhibiting properties of POS also in *R. arrhizus* and it further indicates that **13** is the favored substrate for sterol C14-demethylase in Mucorales (Figure 4).

Surprisingly, the actual amount of ergosterol (**2**) relative to the biomass significantly increased in POS treated *R. arrhizus* hyphae (from 7.07 µg/mg to 7.52 µg/mg). Furthermore, a significant increase of the total sterol content (from 9.26 µg/mg to 12.86 µg/mg) was evident, which is most likely due to the accumulation of new sterols and the increased amount of ergosterol precursors, such as eburicol, which is increased eight-fold when compared to the untreated samples. This finding reflects what was shown by Weete and Wise [26] in propiconazole treated *M. rouxii*. However, this is contrary to our observations in *A. fumigatus*, where inhibition of sterol C14-demethylase resulted in ergosterol reduction (from 9.29 µg/mg to 7.41 µg/mg), and in this course resulted in a decrease of the total sterol content from 9.78 µg/mg to 8.81 µg/mg, although this decrease was not significant. Our results that were obtained for *A. fumigatus* correlate with the results of Alcazar-Fuoli et al. [27], showing that in strains with defective sterol C14-demethylase, caused by mutations in one of the two *cyp51* genes, statistically lower amounts of total ergosterol were detected than in the wildtype strain.

Another sterol, ergosta-5,7-dien-3β-ol (**7**), was clearly reduced from 10.6% to 5.1% in *R. arrhizus*. In *A. fumigatus*, the opposite effect was observed for **7**, resulting in a slight increase from 0.3% to 1.9%. Ergosta-5,7-dien-3β-ol (**7**) is a substrate for sterol C22-desaturase [25], the final enzyme in ergosterol biosynthesis, which converts **7** into ergosterol (**2**). The decrease of non-C14-methylated sterol levels under azole treatment can be explained by an up-regulation of ergosterol biosynthesis enzymes, which is aimed at converting all of the intermediates into ergosterol (e.g., **7**), to avoid the impairment of membrane function. In both, *R. arrhizus* and *A. fumigatus*, a significant accumulation (relative and total amount) of lanosterol (**10**, Figure 1, Figure 2, Figure 3 and Figure 4) was observed. Lanosterol (**10**) is a further physiological substrate for the inhibited enzyme sterol C14-demethylase (Figure 1 and Figure 4), which explains the accumulation due to enzyme inhibition, even though eburciol (**13**) was shown to be the favored enzyme substrate in molds (Mucorales and *A. fumigatus*) [24,25,26,27].

Most interestingly, the non-physiological 14-methylergosta-8,24(28)-diene-3β,6α-diol (**15**) was found (0.7%) in POS that was treated *R. arrhizus.* Accumulation of this toxic intermediate [28,29,46] under the inhibition of sterol C14-demethylase was only detected in yeasts so far, but not in *A. fumigatus* or another filamentous fungus (Figure 4). Further studies are needed to verify if the accumulation of 14-methylergosta-8,24(28)-diene-3β,6α-diol (**15**) is unique for *R. arrhizus* or is ubiquitously found in clinically relevant Mucorales upon azole treatment.

The relative amounts of sterols **1**, **3**–**6**, **8**, **9**, **11**, **12**, **14** were not, or only to a minor extent, affected by POS treatment Table 2.

In conclusion, the azole activity in Mucorales results in significant alterations of the sterol composition, but it does not subsequently lead to a reduction of the total sterol content. The high amount of eburicol, even in untreated hyphae of the Mucorales, let us hypothesize that these fungi could be less affected by the accumulation of non-physiological intermediates due to azole treatment, which is reflected in their lower sensitivity to azole drugs when compared to other molds. In the genome of *R. arrhizus* two paralogous genes encoding for sterol C14-demethylase have been found [20]. It remains to be elucidated if the expression of both the genes is effected to a similar extent in the presence of azoles. So far, our results in *R. arrhizus* let us hypothesize that sterol C14-demethylase is only partly inhibited, as *R. arrhizus* is still able to synthesize ergosterol. This has been shown for other fungi as well, and it reflects that molds inherit a wide repertoire of adaption mechanisms to overcome such drawbacks. From the sterol pattern, we learn that in Mucorales the entry into the post-lanosterol pathway of ergosterol biosynthesis is similar to other molds, whereas under azole treatment, differences to molds, e.g., *A. fumigatus* were detected, resulting in the accumulation of a non-physiological sterol (14-methylergosta-8,24(28)-diene-3β,6α-diol, **15**) which has so far only been found in azole treated yeasts (Figure 4). Because the Mucorales exhibit high variability in their susceptibility to azoles, it is of special interest to decipher the effect of azoles on sterol composition and the content of other clinically relevant mucoralean fungi.

## 3. Material and Methods

### 3.1. Fungal Strains Used in This Study

The following fungal strains, all being obtained from the strain collection of the Division of Hygiene and Medical Microbiology, Medical University Innsbruck (Innsbruck, Austria), were used for sterol analysis: *Aspergillus fumigatus (ATCC46645)*, *Lichtheimia corymbifera* (CBS 109940), *Lichtheimia ramosa* (CBS 101.55), *Mucor circinelloides* (CBS 394.68), *Rhizomucor pusillus* (CBS 219.31), *Rhizopus arrhizus* (CBS 126971), and *Rhizopus microsporus* (CBS 102277).

### 3.2. Fungal Growth and Culture Conditions

All of the strains were cultivated on supplemented minimal agar (SUP) at 37 °C until sporulation (five days). Spores were obtained by harvesting them with sterile spore suspension buffer (0.9% NaCl, 0.01% Tween80). To obtain mycelia for sterol extraction, all of the strains were cultivated in RPMI_1640_ cell culture medium (Sigmaaldrich, Vienna, Austria) at 37 °C with shaking overnight. Cultures of *R. arrhizus* and *R. microsporus* with optimal growth (fine mycelia, no formation of hyphal pellets) were harvested and transferred to a new shake flask containing RPMI_1640_ plus 0.5 µg/mL posaconazole (POS). Cultures were further incubated in the presence of posaconazole for 4 h. Untreated controls were transferred into new medium without posaconazole. Then, the cultures were harvested by filtration, washed, and freeze dried to determine the fungal biomass on dry weight basis.

### 3.3. Sterol Extraction

Six mg of dry fungal biomass were used for sterol extraction as described by Müller et al. [25].

### 3.4. Gas Chromatography-Mass Spectrometry (GC-MS) Analysis of Sterol TMS Ethers

Sterol pattern was determined by GC-MS, according to Müller et al. [25,43]. The quantification, managed with an external calibration with ergosterol, consists of six levels with concentrations up to 20 µg/mg. The base peak of each sterol TMS ether were taken as a quantifier ion for calculating the peak areas for IS cholestane *m*/z 217, **1** ergosta-5,8,22-trien-3β-ol (lichesterol) *m*/*z* 363, **2** ergosta-5,7,22-trien-3β-ol (ergosterol) *m*/*z* 363, **3** ergosta-7,22-dien-3β-ol *m*/*z* 343, **4** ergosta-5,8,22,24(28)-tetraen-3β-ol *m*/*z* 466, **5** ergosta-7,22,24(28)-trien-3β-ol *m*/*z* 343, **6** ergosta-5,7,24(28)-trien-3β-ol *m*/*z* 363, **7** ergosta-5,7-dien-3β-ol *m*/*z* 365, **8** ergosta-7,24(28)-dien-3β-ol (episterol) *m*/*z* 343, **9** ergost-7-en-3β-ol *m*/*z* 472, **10** 4,4,14-trimethylcholesta-8,24-dien-3β-ol (lanosterol) *m*/*z* 393, **11** 4,4-dimethylcholesta-8,24-dien-3β-ol (T-MAS) *m*/*z* 379, **12** 4-methylergost-8-en-3β-ol *m*/*z* 486, **13** 4,4,14-trimethylergosta-8,24(28)-dien-3β-ol (eburicol) *m*/*z* 407, **14** 4,4-dimethylergosta-8,24(28)-dien-3β-ol *m*/*z* 408, and **15** 14-methylergosta-8,24(28)-diene-3β,6α-diol *m*/*z* 377. Sterols 4,4-dimethylcholesta-8,14,24-trien-3β-ol (FF-MAS) 4,4-dimethylergosta-8,14,24(28)-trien-3β-ol, and 14-methylergosta-8,24(28)-dien-3β-ol were not detected in this study. For detailed information about these sterols, see literature [25]. Representative selected ion chromatograms of sterol fractions from *R. arrhizus* are given in Figure 2.

The amount of each sterol was expressed as µg per mg dry weight. The results represent the mean (µg/mg ± S.D.) of two independent biological replicates, including six technical parallels. Experiments involving POS treatment were carried out in triplicate. The sterol composition is expressed as relative amount of total sterols (%).

## Figures and Tables

**Table 1:** Sterol content and sterol composition of six different mucormycete species and *A. fumigatus*. Cultures were grown in RPMI_1640_ medium over night at 37 °C before sterol extraction. The results are presented as the average of two independent experiments, comprising 6 technical replicates in total. Sterol composition is given as the relative amount of the respective sterol (%, in bold letters) of all the sterols detected. Sterol content is expressed as µg sterol intermediate/mg biomass (dry weight). Standard deviation is given in brackets. * n.d. = not detected, ** i.t. = in traces (< 0.04 µg/mg); major sterols are indicated in red.

Compound			Relative [%] and Absolute [µg/mg] Amount of Sterols in Each Species						
No.	IUPAC Name	Common Name	*L. corymbifera*	*L. ramosa*	*M. circinelloides*	*Rh. pusillus*	*R. arrhizus*	*R. microsporus*	*A. fumigatus*
1	ergosta-5,8,22-trien-3β-ol	Lichesterol	**0.2** 0.02 _(± 0.00)_	**0.4** 0.07 _± 0.02)_	**0.3** 0.07 _(± 0.00)_	**0.2** 0.05 _(± 0.00)_	**0.8** 0.07 _(± 0.01_	**0.9** 0.13 _(± 0.02_	**1.2** 0.12 _(± 0.03)_
2	ergosta-5,7,22-trien-3β-ol	Ergosterol	**57.5** 6.45 _(± 0.66)_	**69.2** 13.69 _(± 3.76)_	**65.8** 12.93 _(± 0.37)_	**38.0** 8.13 _(± 0.33)_	**76.3** 7.07 _(± 0.04)_	**70.3** 9.91 _(± 1.85)_	**95.0** 9.29 _(± 2.28)_
3	ergosta-7,22-dien-3β-ol		**3.5** 0.39 _(± 0.04)_	i.t.**	n.d.*	**1.9** 0.41 _(± 0.02)_	**0.2** 0.02 _(± 0.01)_	**0.2** 0.03 _(± 0.02)_	**n.d.**
4	ergosta-5,7,22,24(28)-tetraen-3β-ol		**0.4** 0.04 _(± 0.00)_	**0.6** 0.11 _(± 0.00)_	**0.5** 0.10 _(± 0.01)_	**0.3** 0.06 _(± 0.00)_	**1.0** 0.09_± 0.02)_	**1.2** 0.17 _(± 0.03)_	**0.7** 0.06 _(± 0.01)_
5	ergosta-7,22,24(28)-trien-3β-ol		**0.2** 0.02 _(± 0.01)_	**0.2** 0.04 _(± 0.03)_	**0.2** 0.04 _(± 0.00)_	**0.2** 0.03 _(± 0.02)_	**0.2** 0.03 _± 0.02)_	**0.2** 0.03 _(± 0.02)_	**0.2** 0.02 _(± 0.00)_
6	ergosta-5,7,24(28)-trien-3β-ol		n.d. *	**7.4** 1.50 _(± 1.94)_	**10.4** 2.05 _(± 0.37)_	n.d. **	**2.6** 0.24 _± 0.22)_	**3.3** 0.46 _(± 0.39)_	**0.6** 0.06 _(± 0.01)_
7	ergosta-5,7-dien-3β-ol		**28.5** 3.19 _(± 0.36)_	**12.3** 2.48 _(± 0.11)_	**10.6** 2.07 _(± 0.26)_	**37.2** 9.96 _(± 0.07)_	**10.6** 0.98 _(± 0.19)_	**12.9** 1.82 _(± 0.43)_	**0.3** 0.03 _(± 0.00)_
8	ergosta-7,24(28)-dien-3β-ol	Episterol	**2.4** 0.27 _(± 0.04)_	**1.9** 0.38 _(± 0.13)_	**1.8** 0.35 _(± 0.01)_	**1.4** 0.29 _(± 0.01)_	**2.1** 0.20 _(± 0.13)_	**2.4** 0.34 _(± 0.19)_	**0.8** 0.08 _(± 0.01)_
9	ergost-7-en-3β-ol		**3.7** 0.41 _(± 0.02)_	**0.1** 0.02 _(± 0.03)_	i.t. **	**3.0** 0.64 _(± 0.02)_	**0.3** 0.03 _(± 0.02)_	**0.5** 0.07 _(± 0.03)_	n.d. *
10	4,4,14-trimethylcholesta-8,24-dien-3β-ol	Lanosterol	**0.3** 0.03 _(± 0.00)_	**1.1** 0.21 _(± 0.24)_	**1.3** 0.26 _(± 0.04)_	**0.7** 0.07 _(± 0.03)_	**0.7** 0.07 _(± 0.03)_	**1.0** 0.14 _(± 0.05)_	**0.2** 0.02 _(± 0.00)_
11	4,4-dimethylcholesta-8,24-dien-3β-ol	T-MAS	**0.2** 0.04 _(± 0.00)_	**0.6** 0.12 _(± 0.13)_	**0.7** 0.15 _(± 0.01)_	**0.6** 0.13 _(± 0.01)_	**0.2** 0.02 _(± 0.00)_	**0.2** 0.03 _(± 0.00)_	**0.4** 0.04 _(± 0.00)_
12	4-methylergost-8-en-3β-ol		**0.4** 0.05 _(± 0.00)_	**0.1** 0.01 _(± 0.02)_	i.t. **	**0.9** 0.18 _(± 0.00)_	**0.2** 0.02 _(± 0.02)_	**0.2** 0.02 _(± 0.02)_	n.d. *
13	4,4,14-trimethylergosta-8,24(28)-dien-3β-ol	Eburicol	**2.0** 0.22 _(± 0.01)_	**5.3** 1.07 _(± 1.33)_	**7.2** 1.41 _(± 1.16)_	**15.4** 3.29 _(± 0.18)_	**3.7** 0.34 _(± 0.12)_	**5.5** 0.77 _(± 0.15)_	n.d. *
14	4,4-dimethylergosta-8,24(28)-dien-3β-ol		**0.7** 0.08 _(± 0.01)_	**0.9** 0.17 _(± 0.19)_	**1.1** 0.21 _(± 0.01)_	**0.7** 0.15 _(± 0.00)_	**0.3** 0.03 _(± 0.01)_	**0.3** 0.05 _(± 0.01)_	**0.6** 0.06 _(± 0.00)_
	total sterol content		**100** 11.22 _(± 1.16)_	**100** 20.17 _(± 7.83)_	**100** 19.64 _(± 0.45)_	**100** 21.39 _(± 0.61)_	**100** 9.26 _(± 0.66)_	**100** 14.09 _(± 2.96)_	**100** 9.78 _(± 2.36)_

**Table 2:** Sterol content and sterol composition of *R. arrhizus* and *A. fumigatus* confronted with sublethal concentrations of posaconazole (POS) when compared to the untreated controls. Cultures were grown in RPMI_1640_ medium over night at 37 °C before being transferred to fresh media containing 0.5 µg/mL POS, or no POS. Cultures were incubated for additional 4 h before sterol extraction. The results are presented as the average of two independent experiments, comprising six technical replicates in total. Sterol composition is given as the relative amount of the respective sterol (%, in bold letters) of all sterols detected. Sterol content is expressed as µg sterol intermediate/mg biomass (dry weight). Standard deviation is given in brackets.* n.d. = not detected, ** i.t. = in traces (< 0.04 µg/mg), major sterols are indicated in red.

Compound			Relative [%] and Absolute [µg/mg] Amount of Sterols			
			*R. arrhizus*		*A. fumigatus*	
No.	IUPAC Name	Common Name	POS Treated	Untreated	POS Treated	Untreated
1	ergosta-5,8,22-trien-3β-ol	Lichesterol	**0.7** 0.09 _(± 0.01)_	**0.8** 0.07_(± 0.01)_	**1.2** 0.10 _(± 0.00)_	**1.2** 0.12 _(± 0.03)_
2	ergosta-5,7,22-trien-3β-ol	Ergosterol	**58.5** 7.52 _(± 0.20)_	**76.3** 7.07_(SD ± 0.04)_	**84.1** 7.41 _(± 1.05)_	**95.0** 9.29 _(± 2.28)_
3	ergosta-7,22-dien-3β-ol		**0.1** 0.01 _(± 0.01)_	**0.2** 0.02 _(± 0.01)_	**0.1** 0.01 _(± 0.01)_	n.d. *
4	ergosta-5,7,22,24(28)-tetraen-3β-ol		**0.7** 0.09 _(± 0.02)_	**1.0** 0.09 _(± 0.02)_	**0.8** 0.07 _(± 0.01)_	**0.7** 0.06 _(± 0.01)_
5	ergosta-7,22,24(28)-trien-3β-ol		**0.1** 0.01 _(± 0.00)_	**0.2** 0.03 _(± 0.02)_	**0.2** 0.01 _(± 0.01)_	**0.2** 0.02 _(± 0.00)_
6	ergosta-5,7,24(28)-trien-3β-ol		**0.9** 0.12 _(± 0.12)_	**2.6** 0.24 _(± 0.22)_	**0.6** 0.05 _(± 0.04)_	**0.6** 0.06 _(± 0.01)_
7	ergosta-5,7-dien-3β-ol		**5.1** 0.66 _(± 0.33)_	**10.6** 0.98 _(± 0.19)_	**1.9** 0.17 _(± 0.10)_	**0.3** 0.03 _(± 0.00)_
8	ergosta-7,24(28)-dien-3β-ol	Episterol	**0.8** 0.10 _(± 0.00)_	**2.1** 0.20 _(± 0.13)_	**1.0** 0.09 _(± 0.02)_	**0.8** 0.08 _(± 0.01)_
9	ergost-7-en-3β-ol		**0.1** 0.01_(± 0.01)_	**0.3** 0.03 _(± 0.02)_	i.t. **	n.d. *
10	4,4,14-trimethylcholesta-8,24-dien-3β-ol	Lanosterol	**2.5** 0.33 _(± 0.26)_	**0.7** 0.07 _(± 0.03)_	**1.3** 0.12 _(± 0.12)_	**0.2** 0.02 _(± 0.00)_
11	4,4-dimethylcholesta-8,24-dien-3β-ol	T-MAS	i.t. **	**0.2** 0.02 _(± 0.00)_	**0.4** 0.04 _(± 0.00)_	**0.4** 0.04 _(± 0.00)_
12	4-methylergost-8-en-3β-ol		**0.1** 0.01_(± 0.01)_	**0.2** 0.02 _(± 0.02)_	n.d. *	n.d. *
13	4,4,14-trimethylergosta-8,24(28)-dien-3β-ol	Eburicol	**29.1** 3.74 _(± 1.58)_	**3.7** 0.34 _(± 0.12)_	**6.9** 0.61 _(± 0.80)_	n.d.*
14	4,4-dimethylergosta-8,24(28)-dien-3β-ol		i.t. **	**0.3** 0.03 _(± 0.01)_	**0.7** 0.06 _(± 0.02)_	**0.6** 0.06 _(SD ± 0.00)_
15	14-methylergosta-8,24(28)-diene-3β,6α-diol		**0.7** 0.09 _(± 0.11)_	n.d. *	n.d. *	n.d.*
	**total sterol content**		**100** 12.86 _(± 1.30)_	**100** 9.26 _(± 0.66)_	**100** 8.81 _(± 0.08)_	**100** 9.78 _(± 2.36)_
